# Tumor suppressive miR-6775-3p inhibits ESCC progression through forming a positive feedback loop with p53 via MAGE-A family proteins

**DOI:** 10.1038/s41419-018-1119-3

**Published:** 2018-10-17

**Authors:** Lingjiao Meng, Fei Liu, Yingchao Ju, Pingan Ding, Sihua Liu, Sheng Chang, Shina Liu, Yi Zhang, Yishui Lian, Lina Gu, Xiaochong Zhang, Meixiang Sang

**Affiliations:** 1 Research Center, The Fourth Hospital of Hebei Medical University, 050017 Shijiazhuang, Hebei People’s Republic of China; 2 Animal Center, The Fourth Hospital of Hebei Medical University, 050017 Shijiazhuang, Hebei People’s Republic of China; 3 Department of Mathematics, Hebei University of Science and Technology, 050018 Shijiazhuang, People’s Republic of China; 4 Tumor Research Institute, The Fourth Hospital of Hebei Medical University, 050017 Shijiazhuang, Hebei People’s Republic of China

## Abstract

Accumulating evidences indicate that microRNAs (miRNAs) play vital roles in multiple diseases, including cancer. In the present study, we showed that miR-6775-3p plays a tumor suppressive role in esophageal squamous cell carcinoma (ESCC). High expression miR-6775-3p is associated with good clinical outcomes of ESCC patients. Over-expression of miR-6775-3p inhibited tumor growth and liver metastasis of ESCC xenograft tumors. Enforced expression of miR-6775-3p inhibited ESCC cell proliferation, migration, and invasion. KEGG pathway analysis revealed that miR-6775-3p was associated with the genes on “pathway in cancer”. Mechanically, miR-6775-3p inhibited the expression of tumor antigens MAGE-A family through direct binding the 3′UTR region of MAGE-A mRNAs, and attenuated MAGE-A-inhibited transcriptional activity of tumor suppressor p53. In addition, miR-6775-3p also directly inhibits its host gene SLC7A5 which has been reported to play oncogenic roles in cancer progression. Interestingly, miR-6775-3p and its host gene SLC7A5 were directly transcriptionally induced by p53. Thus, for the first time, our study proposed a novel positive feedback regulation between miR-6775-3p and p53 via MAGE-A family, which plays crucial role in ESCC progression.

## Introduction

As a common malignant tumor, esophageal cancer ranks sixth in the cause of cancer-relevant death all over the world^1^. Esophageal squamous cell carcinoma (ESCC) is the main histopathological type of esophageal cancer^2^. Although ESCC treatment options including radiotherapy, chemotherapy and surgery have made great progress, the overall 5-year survival rate of patients with ESCC remains unfavorable^3^. Despite many oncoproteins have been found participating in the progress of ESCC, more and more studies inveal that there are other species of biological molecules such as non-coding RNA (ncRNA) playing vital roles in the process^4^.

MicroRNAs (miRNAs) are a type of endogenous ncRNAs that modulate gene expression through inhibiting translation or cleaving RNA transcripts by a sequence-specific way^5,6^. It is well established that miRNAs involve in almost all physiological and pathological processes, and play crucial role in cancer progression^7–9^. Indeed, aberrant miRNA expression was observed in human cancers, with evidence for a causative role in tumorigenesis^10,11^. In human, there are about half of miRNA genes locate among independent transcription units, while the intragenic miRNAs are embedded within intronic regions and oriented on the same DNA strand of host genes^12^. Intergenic miRNAs are transcribed from respective transcript units, like other protein-coding genes, by RNA polymerase II and cleaved then processed to generate mature miRNAs^13^. Intronic miRNAs are considered to be processed from the introns of the hosting transcription units, therefore sharing universal regulatory mechanisms and expression patterns with their host gene^14,15^. Notably, a variety of well-known transcription factors, such as c-Myc and CREB, have been found to regulate mRNA and miRNA expression^16,17^.

In this study, we found that high expression miR-6775-3p is related to the more favorable prognosis of ESCC patients. Over-expression of miR-6775-3p inhibited the growth of ESCC xenograft tumors and liver metastasis. Enforced expression of miR-6775-3p inhibited ESCC cell proliferation, migration, and invasion, while downregulation of miR-6775-3p increased ESCC cells proliferation, migration and invasion. Mechanically, miR-6775-3p inhibited the expression of tumor antigens MAGE-A family through direct binding the 3′UTR region of MAGE-A mRNAs, and attenuated MAGE-A-inhibited transcriptional activity of tumor suppressor p53. In addition, miR-6775-3p also directly inhibits its host gene SLC7A5 which has been found to function as an oncogene in tumor progression. Interestingly, miR-6775-3p and its host gene SLC7A5 were directly transcriptionally induced by p53. Thus, for the first time, our study proposed a novel positive feedback regulation between miR-6775-3p and p53 via MAGE-A family, which plays crucial role in ESCC progression.

## Materials and methods

### Clinical specimens

We chose 138 specimens of patients with primary ESCC in our hospital from January 2011 to December 2011. All above patients were esophagoscopy bite-tested pathologically confirmed as esophageal squamous cell carcinoma, and no radiotherapy or chemotherapy before surgery. They were tracked 12–60 months. All patients signed informed consent and the study protocol was approved by the Medical Ethics Committee of our hospital.

### Animal experiment

All animal experiments were authorized by the animal Care Committee of the Fourth Hospital of Hebei Medical University. In xenograft tumor model, four-week-old BALB/c nude mice were applied for the experiment and divided into two groups at random (*n* = 10 for each group). TE1 cells (5 × 10^6^ cells per mouse) transfected with 200 nM miR-6775-3p agomir or NC agomir were subcutaneously injected into the mice. About 1 week later, when the tumor volume reached 100 mm^3^, miR-6775-3p agomir (totally 5 nmol per mouse) or NC agomir (totally 5 nmol per mouse) was injected into the tumor per 3 days. At the same time, tumor volume was recorded. A month later, mice were sacrificed. Tumor weight and some related gene expression were examined. For in vivo metastasis experiment, four-week-old BALB/c nude mice were applied similarly and they were randomly assigned to two groups (*n* = 10 for each group). The mice spleen was exposed to the surgical field after anesthesia. Similarly, TE1 cells transfected with 200 nM miR-6775-3p agomir or NC agomir (5 × 10^6^ cells per mouse) were slowly injected under the splenic capsule of BALB/c nude mice. Five minutes after injection, the spleen was removed. Three days later, miR-6775-3p agomir (totally 8 nmol per mouse) or NC agomir (totally 8 nmol per mouse) was injected into BALB/c nude mice via tail vein twice/week. Two months later, mice were sacrificed and the livers were initially examined by the naked eye, and HE staining was performed. The metastatic counts of each slice were observed.

### HE staining and immunohistochemistry (IHC)

HE staining and IHC were performed on the basis of normal method. Mouse MAGE-A 6C1 polyclonal antibody (Santa Cruz, USA), Rabbit polyclonal Ki67 antibody (Abcam, USA), Rabbit polyclonal PCNA antibody (Abcam, USA), Rabbit polyclonal MMP2 antibody (Abcam, USA), Rabbit polyclonal MMP9 antibody (Abcam, USA), Rabbit polyclonal SLC7A5 antibody (Abcam, USA) were applied for IHC analysis. The immunoreactivity was assessed according to the normal histological method.

### Cell culture

Esophageal cancer cell lines (TE1, Eca109, Ec9706, KYSE30) were cultured in RPMI1640 (GIBCO, USA) added 10% fetal bovine serum (GIBCO, USA), 100 U/ml penicillin and 100 μg/ml streptomycin. Cells were cultivated at 37 °C, 5% CO_2_ volume fraction of the thermostat.

### Oligonucleotide transfection

miRNA mimics, inhibitor and agomir were synthesized by GenePharma (Suzhou, China). The miRNA mimics and inhibitor were used to transfect cells in vitro. The agomir applied for the animal experiment in vivo was methylated and terminal cholesterol-labeled based on miRNA mimics. The sequences are shown in Supplementary Table S1.

### MTT assay

Cells were cultured at a density of 5 × 10^3^ cells/well. After transfection, MTT assays were performed at 0, 24, 48, 72, and 96 h. Ten microliter MTT reagent was added to each well. After incubation at 37 °C for 2 h, the optical density of 450 nm were read out at 570 nm by the microplate reader (Tecan, USA). The above experiment was repeated three times.

### Colony formation assay

Cells were cultivated in 6-well plates at a density of 3 × 10^3^ cells per well. After 1 week, cells were fixed with 4% formaldehyde and stained with Giemsa. The cell colonies were analyzed by the microscope. The colonies which total numbers of individuals are more than 50 were counted. The above experiment was repeated three times. The above assay was performed three times independently.

### Wound healing assay

The transfected ESCC cells were collected and adjusted the density to 5 × 10^5^ /ml. Then we took 2 ml cell suspension and incubated them in 6-well plates with marked five parallel lines on the back. Cells were scraped with 200 μl pipette tips after attachment, then washed twice with PBS, and 2 ml of RPMI 1640 medium without fetal bovine serum was added. The distances from the cells to the middle of the scratches were observed under inverted microscope at 0 and 24 h. Three parallel holes for each experiment were set up and there were three absolute individuals completely.

### Transwell migration and invasion assay

The cell suspensions with fetal bovine serum-free RPMI 1640 culture of various transfected groups (2 × 10^5^/ml) were collected and respectively added to the upper chamber of the Transwell chamber (BD Science, Bedford, MA, USA), and the lower chamber were added 600 μl of RPMI 1640 culture medium containing 10% fetal bovine serum. For transwell invasion assay, the upper chamber was covered with matrigel (BD Science, Bedford, MA, USA) and coagulated overnight. The remaining operation was the same as above. After routinely cultured for 48 h, the upper chamber cells were wiped with a cotton swab and stained with crystal violet dye. The migration and invasion cells were observed and counted under five fields at random. The experiment was duplicated three times.

### RNA extraction and qRT-PCR

Total RNA from cells and tissues were isolated by TRIzol^®^ Reagent (Invitrogen). The cDNA was prepared from above total RNA according to GoScript^TM^ Reverse Transcription System (Promega, USA) protocol. qRT-PCR analysis was operated by GoTaq^®^ qPCR Master Mix (Promega). The primers used were shown in Supplementary Table S1. Bulge-loop miRNA qRT-PCR primer sets (one RT primers for each set) specific for miR-6775-3p were synthesized by RiboBio, Guangzhou, China. GAPDH was applied for mRNA input, and U6 was for miRNA input. The relative expression levels were computed by the comparative *C*_T_ value (ΔΔ*C*_T_).

### Western blot

Western blot analysis was performed according to standard method. Proteins were extracted by RIPA lysis buffer (solarbio, life sciences). After denaturation, proteins were discreted by 10% SDS-PAGE electrophoresis and were transferred onto polyvinylidene fluoride (Millipore, Billerica, MA, USA). Mouse MAGE-A 6C1 antibody (Santa Cruz, USA), and Rabbit SLC7A5 antibody (Abcam, USA) were applied for immunoreactivity. The PVDF membranes were imaged using the ECL Plus (solarbio, life sciences).

### Subcellular fractionation

The subcellular fractionation was prepared as the PARIS^TM^ Kit (Life, USA) protocol. The separate nuclear and cytoplasmic lysate were applied for WB analysis using the mouse MAGE-A 6C1 polyclonal antibody (Santa Cruz, USA), mouse p53 monoclonal antibody (Santa Cruz, USA), Rabbit polyclonal antibody (Abcam, USA), and rabbit monoclonal anti-Lamin B (Abcam, USA) antibody.

### Immunoprecipitation analysis

The whole cell lysates were pre-cleared by incubation with protein G-Sepharose beads (Amersham Pharmacia Biotech) at 4 °C for 1 h. The supernatant was collected after short centrifugation, and then incubated with p53 antibody or normal IgG antibody at 4 °C for 2 h. The immune compounds were precipitated with protein G-Sepharose beads at 4 °C for 1 h, and the non-specific bound proteins were got rid of by washing the beads with the lysis buffer three times at 4 °C. The precipitated proteins were eluted with boiling 1 × SDS sample buffer, separated by 10% SDS-PAGE electrophoresis and analyzed by incubating with anti-MAGE-A or anti-p53 antibody.

### Chromatin immunoprecipitation (ChIP)

ChIP assays was operated as characterized in the datasheet of EZ-Magna ChIP™ G Chromatin Immunoprecipitation Kit (Millipore). Immunoprecipitations were performed with 2 µg of p53 antibody or control IgG. The primers used in this study to detect the enrichment of SLC7A5/miR-6775 promoter DNA sequences are as following, forward, 5′-AGTATCTGTGTGACCTCCGC-3′; reverse, 5′-TGGGTGTTCACAGCAAGACA-3′. The enrichment fold of ChIP DNA was assessed as percentage of normal input.

### Luciferase reporter assay

Cells were cultured at a density of 5 × 10^4^/well in 12-well plate. They were co-transfected with miR-6775-3p mimics or miR-NC and MAGE-A 3′UTR or MAGE-A mut 3′UTR plasmid. Forty-eight hours after transfection, renilla and firefly luciferase activity was measured by dual-luciferase reporter assay kit (Promega, Madison, WI, USA). Renilla luciferase activity was standardized to firefly activity and the ratio was used to be relative activity. For promoter activities measurement, cells were co-transfected with miR-6775/SLC7A5-luc, pRL-SV40 renilla luciferase reporter, GFP-P53 plasmid or empty vector. The remaining operation is the same as above and all the assays were triplicated at least.

### Statistical analysis

Statistical analysis was performed by SPSS 22.0 software. The data were presented as Mean ± SD, and measured by Student’s *t*-test. The association among the cliniopathological parameters were analyzed by Chi-Square test. Kaplan–Meier method was used to evaluate the overall survival, and the log-rank test was performed to estimate differences among various groups. The significance of data was expressed by *P*-values, and *P* < 0.05 was deemed statistically significant.

## Results

### miR-6775-3p is associated with good prognosis of ESCC and inhibits ESCC tumor growth and metastasis in vivo

**Fig. 1:**
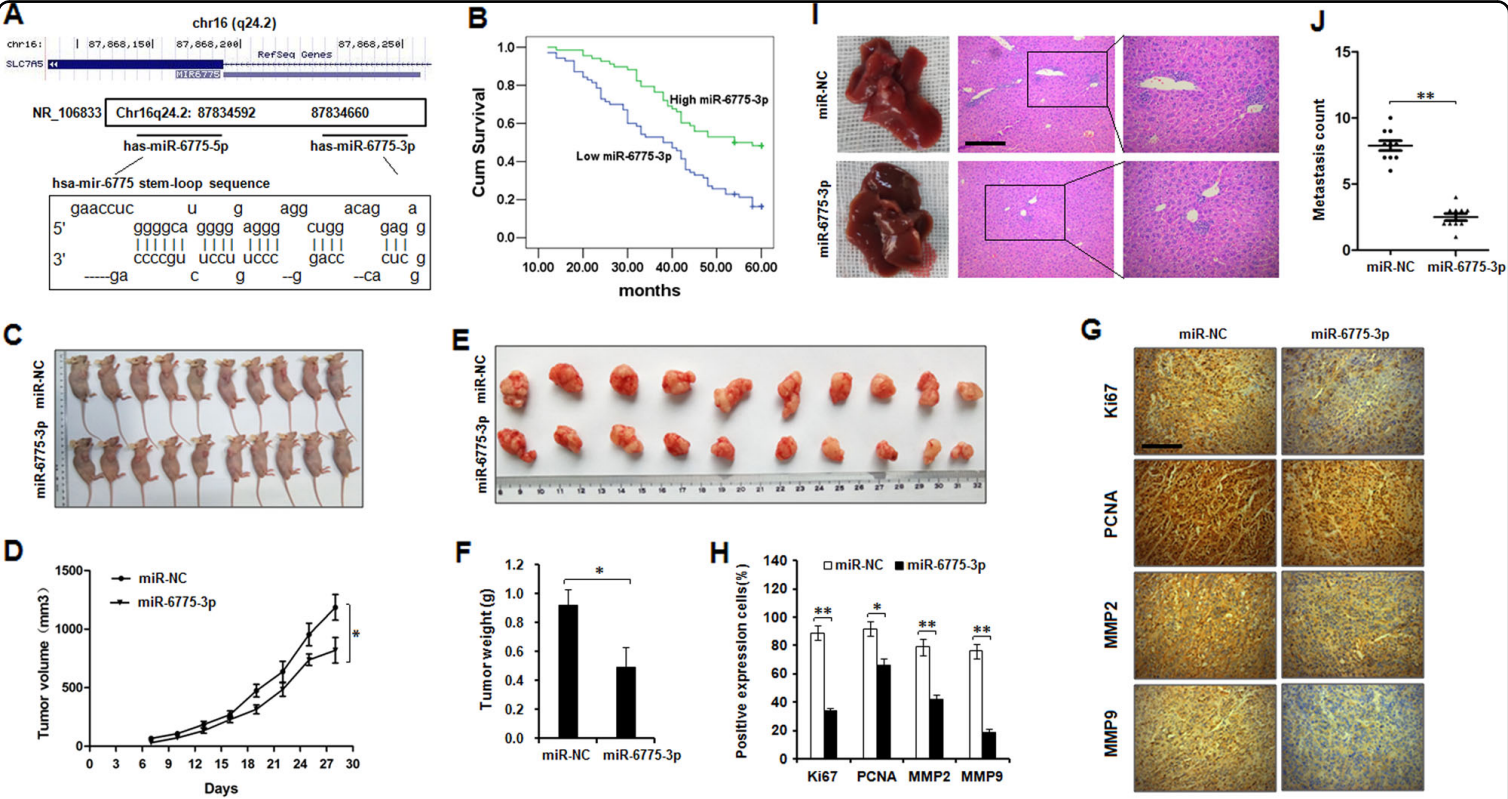
**miR-6775-3p is associated with good prognosis of ESCC and inhibits ESCC tumor growth and metastasis in vivo.** **a** Gene structure of miR-6775 in the chr16 (q24.2) region of human genome. **b** Kaplan–Meier analysis revealed that high expression of miR-6775-3p is associated with better survival of ESCC patients. **c**–**f**, TE1 cells (5 × 10^6^ cells per mouse, *n* = 10 for each group) transfected with miR-6775-3p agomir or NC agomir transfection were subcutaneously injected into BALB/c nude mice. After cell injection, tumors were injected with miR-6775-3p agomir (totally 5 nM per mouse) or NC agomir (totally 5 nM per mouse) per 2 days. Tumor growth and tumor weight were analyzed. *P < 0.05. **g**, **h**, The expression of Ki67, PCNA, MMP2 and MMP9 in mice tumor tissues was examined by IHC. Bars: 50 μm. **P < 0.01, *P < 0.05. **i**, **j** TE1 cells (5 × 10^6^ cells per mouse, *n* = 10 for each group) transfected with miR-6775-3p agomir or NC agomir transfection were slowly injected under the splenic capsule of BALB/c nude mice. Five minutes after injection, the spleen was removed. Three weeks later, mice were sacrificed and the livers were initially examined by the naked eye, and HE staining was performed. The metastatic counts of each slice were observed. Bars: (left) 100 μm; (right) 50 μm. **P < 0.01

We presented the gene structure of miR-6775 in the chr16 (q24.2) region of human genome (Fig 1a). The miR-6775 is nested in an intron of SLC7A5. To confirm the possible function of miR-6775-3p in ESCC progression, we firstly performed qRT-PCR for miR-6775-3p on 138 cases of human ESCC specimens. Our results showed that miR-6775-3p expression was negatively associated with the histological grade, tumor infiltration, lymph node metastasis, distant metastasis or recurrence of ESCC patients (Supplementary Table S2). In addition, lower expression of miR-6775-3p was related to poor prognosis of ESCC patients (Fig. 1b). To investigate the role of miR-6775-3p in tumorigenesis, we detected its expression in four ESCC cell lines (Supplementary Figure S1), and performed over-expression of TE1 (low miR-6775-3p) nude mice xenograft study by using miR-6775-3p agomir or NC agomir transfection system. The decreased tumor growth and tumor weight were found in miR-6775-3p agomir-transfected group as compared with the control group (Fig. 1c–f). We also detected the proliferation-related molecules (Ki67 and PCNA) and the metastatic-related markers (MMP2 and MMP9) in the xenograft tumor tissues by Immunohistochemical staining. The results suggested that these markers were decreased in miR-6775-3p over-expression group (Fig 1g, h). In addition, we also established the TE1 cells liver-metastasis model via injection under the splenic capsule. The results showed that the miR-6775-3p-transfected nude mice formed fewer liver metastasis colonies than those treated with the control miRNA (Fig. 1i, j). Taken together, these results demonstrated that miR-6775-3p probably plays a tumor repressive role in ESCC.

### Effects of miR-6775-3p on the phenotypes of ESCC cells

**Fig. 2:**
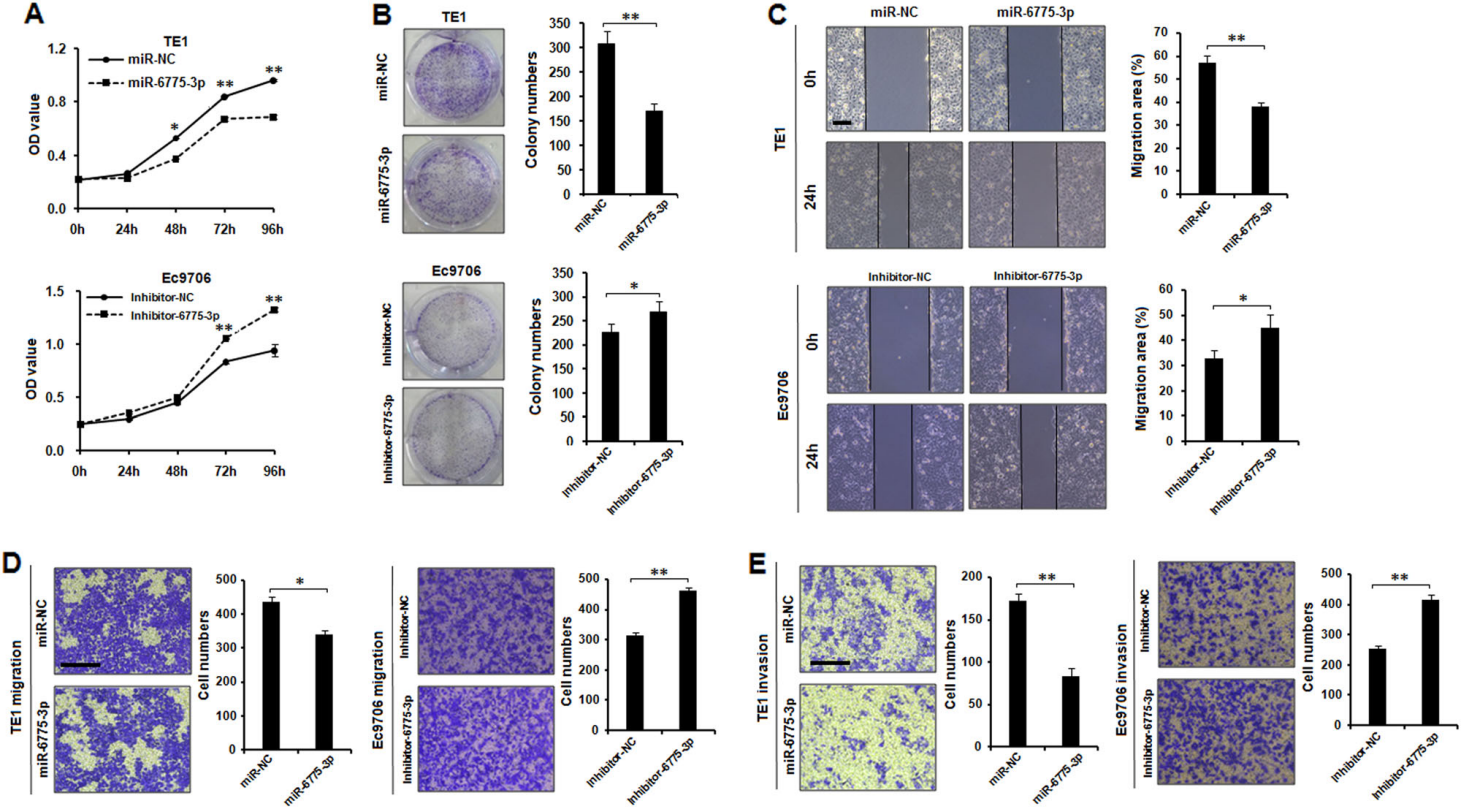
**Effects of miR-6775-3p on the phenotypes of ESCC cells.** **a** Cell proliferation ability of TE1 cells transfected with miR-6775-3p mimics or miR-NC and Ec9706 cells transfected with miR-6775-3p inhibitor or inhibitor-NC were evaluated by MTT assay. *P < 0.05, **P < 0.01. **b** Colony formation ability of TE1 cells transfected with miR-6775-3p mimics or miR-NC and Ec9706 cells transfected with miR-6775-3p inhibitor or inhibitor-NC were evaluated by colony formation assay. *P < 0.05, **P < 0.01. **c** Cell migration abilities of TE1 cells transfected with miR-6775-3p mimics or miR-NC and Ec9706 cells transfected with miR-6775-3p inhibitor or inhibitor-NC were evaluated by wound healing experiment. *P < 0.05, **P < 0.01. Bars: 40 μm. **d**, **e**, Cell migration and invasion abilities of TE1 cells transfected with miR-6775-3p mimics or miR-NC and Ec9706 cells transfected with miR-6775-3p inhibitor or inhibitor-NC were evaluated by transwell migration and matrigel invasion assay. Bars:100μm. *P < 0.05, **P < 0.01

To further investigate the inhibitory mechanism of miR-6775-3p on ESCC initiation and progression, we performed over-expression or down-regulation studies in TE1 or Ec9706 cells by using mature miR-6775-3p mimics or miR-6775-3p inhibitor transfection. The MTT assay and colony formation assay demonstrated that cell proliferation and colony formation were inhibited in miR-6775-3p mimics transfected TE1 cells, whereas enhanced in miR-6775-3p inhibitor transfected Ec9706 cells (Fig. 2a, b). The wound healing assay revealed that over-expression of miR-6775-3p inhibited cell migration of TE1 cells, while miR-6775-3p inhibitor promoted Ec9706 cell migration (Fig. 2c). In addition, over-expression of miR-6775-3p also inhibited migration and invasion of TE1 cells in transwell migration and invasion assays (Fig. 2d, e). However, miR-6775-3p inhibitor enhanced migration and invasion of Ec9706 cells in transwell migration and matrigel invasion assay (Fig. 2d, e). Our fondings revealed that miR-6775-3p inhibited ESCC progression through suppressing cell proliferation, migration and invasion.

### Identification of miR-6775-3p-mediated molecular pathways

To gain further insight into the molecular mechanisms and pathways regulated by miR-6775-3p, we obtained the putative target genes regulated by miR-6775-3p through searching the TargetScan database. According to the results, a total of 2755 targets, with 4056 sites were deposited in the database. GENECODIS 3.0 program was used to analyze and characterize the genes above in KEGG pathway. Supplementary Table S3 showed the top 14 pathways that were associated with miR-6775-3p target genes. In these pathways, “pathways in cancer” was most significantly associated with miR-6775-3p associated pathways, and 63 genes were included in this pathway. Most of these genes are shown to play oncogenic roles in cancer progression. These data suggested that miR-6775-3p was the potential tumor repressive miRNA.

### Tumor antigens MAGE-A family members (including MAGE-A1, MAGE-A2, MAGE-A3, MAGE-A6, MAGE-A8, MAGE-A9, MAGE-A10, MAGE-A11, and MAGE-A12) are direct targets of miR-6775-3p

**Fig. 3:**
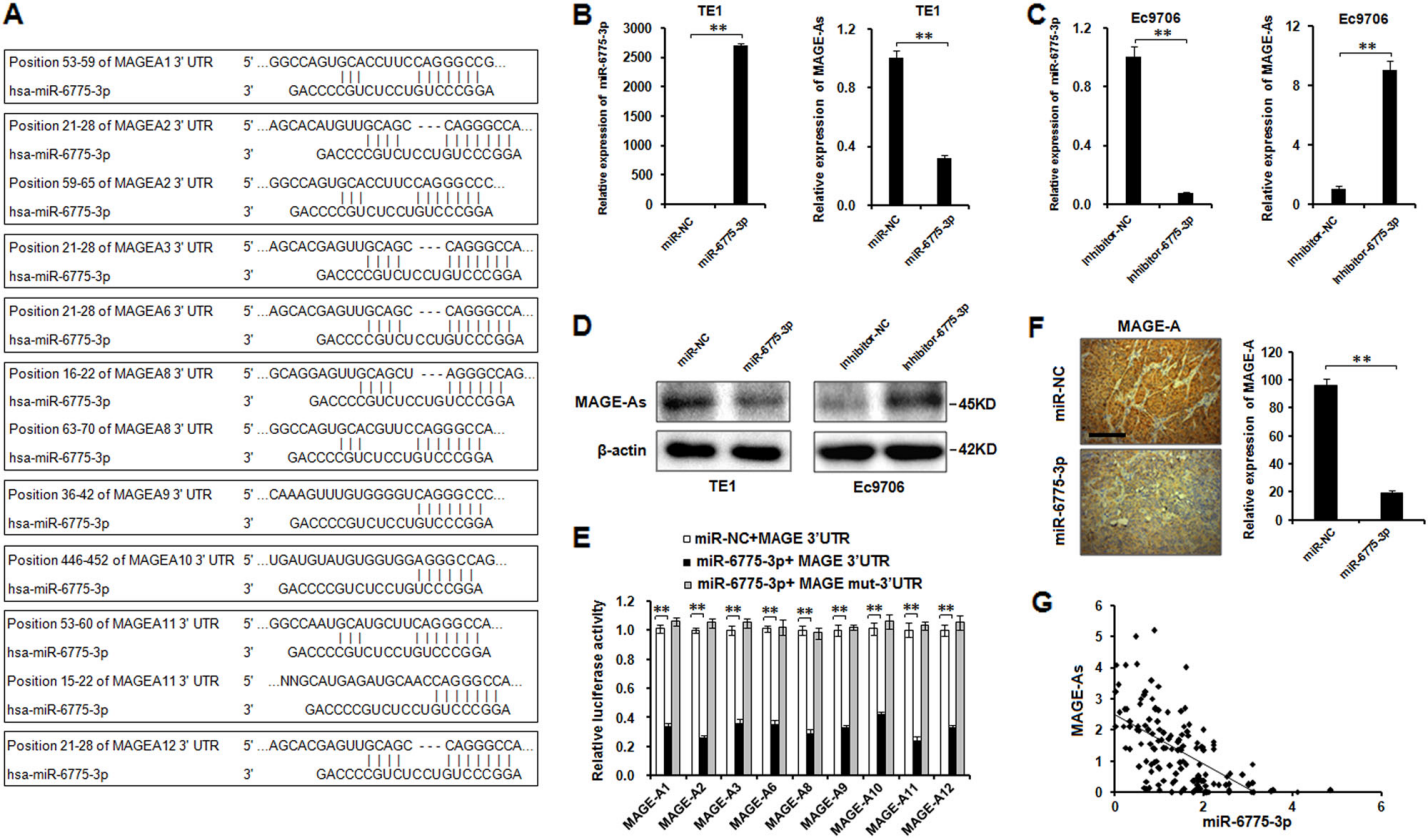
**Tumor antigens MAGE-A family members (including MAGE-A1, MAGE-A2, MAGE-A3, MAGE-A6, MAGE-A8, MAGE-A9, MAGE-A10, MAGE-A11, MAGE-A12) are direct targets of miR-6775-3p.** **a** The binding sites of miR-6775-3p with the 3′UTR of MAGE-A family members. **b** Expression of miR-6775-3p and MAGE-As in TE1 cells transfected with miR-6775-3p mimics or miR-NC, detected by qRT-PCR. **P < 0.01. **c** Expression of miR-6775-3p and MAGE-As in Ec9706 cells transfected with miR-6775-3p inhibitor or inhibitor-NC, detected by qRT-PCR. **P < 0.01. **d** Expression of MAGE-As in TE1 cells transfected with miR-6775-3p mimics or miR-NC and Ec9706 cells transfected with miR-6775-3p inhibitor or inhibitor-NC, detected by western blot. **e** Luciferase reporter assays for luciferase activity of luc-MAGE-A 3′UTRs or mutated luc-MAGE-A 3′UTRs in TE1 cells co-transfected with miR-6775-3p mimics. **P < 0.01. **f** The expression of MAGE-As in mice tumor tissues was examined by IHC. **P < 0.01. Bars: 50 μm. **g** Expression correlation between miR-6775-3p and MAGE-As detected by qRT-PCR

According to the TargetScan database, we found that there exists at least one miR-6775-3p binding site on the 3′UTR region of tumor antigen MAGE-A family (including MAGE-A1, MAGE-A2, MAGE-A3, MAGE-A6, MAGE-A8, MAGE-A9, MAGE-A10, MAGE-A11, and MAGE-A12), which plays oncogenic role in ESCC progression (Fig. 3a)^18^. We then performed qRT-PCR and western blot to investigate whether MAGE-A family members were the direct targets of miR-6775-3p. Because of the high homology of MAGE-A family members, we designed the primers (termed as MAGE-As) which can detect the majority of MAGE-A family members. At the protein level, we used MAGE-A 6C1 antibody to detect the majority of MAGE-A proteins. The qRT-PCR results revealed that MAGE-A family expression was markedly repressed in miR-6775-3p-overexpressed TE1 cells, and increased in miR-6775-3p-suppressed Ec9706 cells (Fig. 3b, c). Similar results were observed at protein level (Fig. 3d).

To further confirm the MAGE-A genes as direct targets of miR-6775-3p, we constructed wild-type 3′UTR and miR-6775-3p seed region-mutagenized 3′UTR region of each MAGE-A family members into a *Renillta* luciferase reporter vector. The luciferase reporter assay results showed that miR-6775-3p decreased the luciferse activity of wild-type MAGE-A 3′UTRs, while it had almost no effect on the luciferase activity of mutagenized MAGE-A 3′UTRs (Fig. 3e). We also detected the MAGE-A expression in miR-6775-3p agomir-transfected or NC agomir-transfected TE1 mice xenograft tissues, and found that the expression of MAGE-A was significantly inhibited in miR-6775-3p agomir-transfected group (Fig. 3f). In human ESCC tissues, a negative correlation was found between miR-6775-3p and MAGE-A expression (Fig. 3g). These results suggested that MAGE-A family members are direct targets of miR-6775-3p.

### miR-6775-3p inhibits the proliferation, migration, and invasion of ESCC cells by direct targeting MAGE-A

**Fig. 4:**
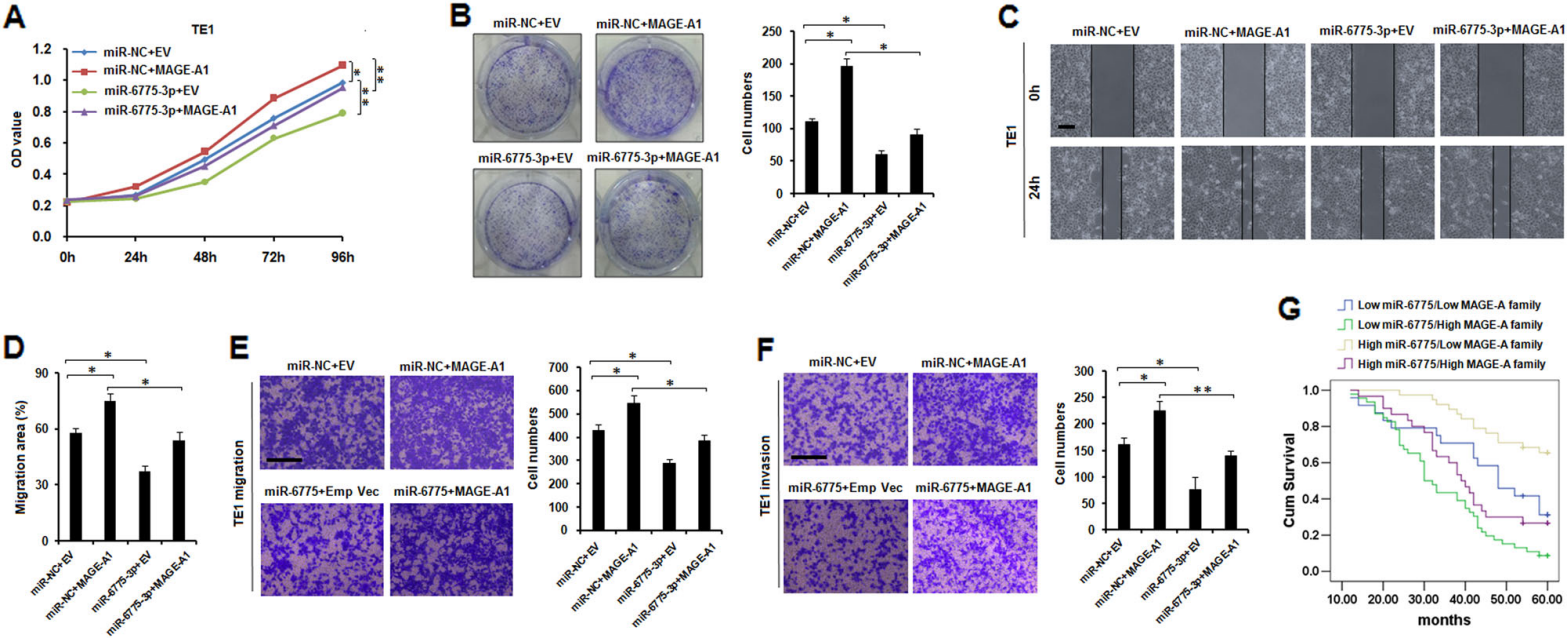
**miR-6775-3p inhibits the proliferation, migration and invasion of ESCC cells by direct targeting MAGE-As.** **a** MTT assay showed that miR-6775-3p suppressed cell proliferation of TE1 cells. After co-transfeced with miR-6775-3p and MAGE-A1, the suppressive effect of miR-6775-3p was reversed. *P < 0.05, **P < 0.01. **b** Colony formation assay showed that miR-6775-3p suppressed the colony formation of TE1 cells. After co-transfeced with miR-6775-3p mimics and MAGE-A1, the suppressive effect of miR-6775-3p was reversed. *P < 0.05. **c**, **d** Wound healing experiment showed that miR-6775-3p suppressed the migration of TE1 cells. After co-transfeced with miR-6775-3p mimics and MAGE-A1, the suppressive effect of miR-6775-3p was reversed. Bars:50μm. *P < 0.05. **e**, **f** Transwell migration and matrigel invasion assay showed that miR-6775-3p suppressed cell migration and invasion abilities of TE1 cells. After co-transfeced with miR-6775-3p mimics and MAGE-A1, the suppressive effect of miR-6775-3p was reversed. Bars: 100 μm. *P < 0.05, **P < 0.01. **g** Kaplan–Meier survival analysis showed that ESCC patients with low miR-6775-3p expression and high expression of MAGE-A family had poor clinical outcomes, and the patients with high miR-6775-3p expression and low expression of MAGE-A family had better clinical outcomes, as compared with other patients

To confirm whether miR-6775-3p inhibits ESCC cell proliferation, migration, and invasion through targeting MAGE-A family, TE1 cells were co-transfected with miR-6775-3p mimics plus MAGE-A1 over-expression plasmids, and cell migration and invasion were evaluated. According to the results in Fig. 4a, b, the inhibitory effects of miR-6775-3p on cell proliferation and colony formation were drastically attenuated in the presence of MAGE-A1. Similarly, the inhibitory effects of miR-6775-3p on cell migration and invasion were also rescued in the presence of MAGE-A1 (Fig. 4c–f). Similar results were obtained in MAGE-A9-transfected condition (Supplementary Figure S2). In addition, ESCC patients who had low miR-6775-3p expression and high expression of MAGE-A family showed worse clinical outcomes, and the patients who had high miR-6775-3p expression and low expression of MAGE-A family showed better clinical outcomes, as compared with other patients (Fig 4g). Our results indicated that miR-6775-3p inhibits ESCC cell proliferation, migration and invasion by direct targeting MAGE-A family.

### miR-6775-3p attenuates MAGE-A-inhibited transcriptional activity of p53

**Fig. 5:**
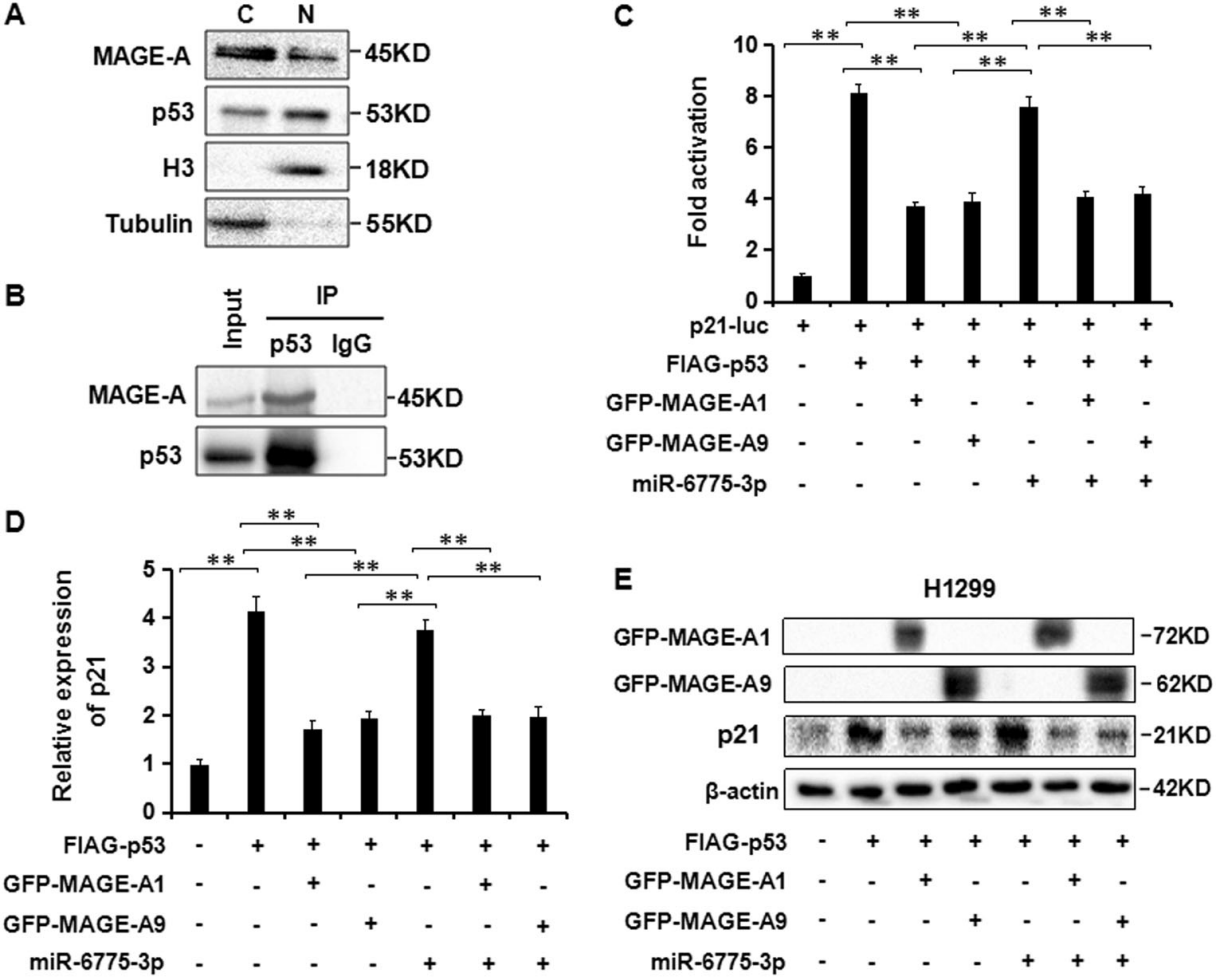
**miR-6775-3p attenuates MAGE-A-inhibited transcriptional activity of p53.** **a** Subcellular localization of p53 and MAGE-A proteins in Eca109 cells was detected by Western blot. C (Cytoplasm); N (Nuclear). **b** The complex formation of p53 and MAGE-A proteins was detected by immunoprecipitation in Eca109 cells. **c** Luciferase reporter assays for luciferase activity of luc-p21^WAF1^ promoter in H1299 cells co-transfected with combination of different plasmids. **P < 0.01. **d** The expression of p21 in H1299 cells co-transfected with combination of different plasmids was examined by qRT-PCR. **P < 0.01. **e** The expression of p21 in H1299 cells co-transfected with combination of different plasmids was examined by western blot. **P < 0.01

The interaction between MAGE-A proteins with p53 has been reported to occlude the binding of p53 with p53-responsive promoters, lead to the decreased p53-dependent transcription^19–21^. In addition, Shyamal et al. reported that inhibition of MAGE-A by miR-34a resulted in increased expression of p53, and a novel positive feedback loop was established between miR-34a and p53 for the first time through the MAGE-A proteins^22^. Therefore, we examined whether the interaction between MAGE-A1 and p53 could affect the transcription of p53, and whether miR-6775-3p could affect the transcriptional activity of p53 through targeting MAGE-A family. Firstly, sub-cellular localization of p53 and MAGE-A were detected in p53-wild type Eca109 cells. The immunoblotting results showed that MAGE-A and p53 were co-localized in both cell cytoplasm and nuclear (Fig 5a). We then performed the immunoprecipitaion in Eca109 cells. Our results showed that p53 could pull down the MAGE-A proteins in cells (Fig. 5b). The luciferase reporter assay results in p53-deficient H1299 cells which express endogenous MAGE-A demonstrated that MAGE-A1 inhibited the p53-mediated up-regulation of luciferase activity driven by p21^WAF1^ promoter, while miR-6775-3p abolished the MAGE-A1-mediated inhibition of p21^WAF1^ promoter luciferase activity. However, cotransfection of MAGE-A1 deprived of the 3′UTR with miR-6775-3p abrogated the enhanced p21^WAF1^ promoter luciferase activity (Fig 5c). In supporting with the luciferase reporter assay results, enforced expression of MAGE-A1 in H1299 cells reduced p53-mediated up-regulation of endogenous p21^WAF1^ expression, whereas miR-6775-3p reversed the reduction of endogenous p21^WAF1^ expression by targeting endogenous MAGE-A1, and P21^WAF1^ expression dropped to the former level after adding exogenous MAGE-A1 without the 3′UTR (Fig. 5d, e). Similar results were obtained in MAGE-A9-transfected H1299 cells (Fig. 5c–e). Taken together, our results supported that MAGE-A family proteins inhibited the transcription activity of p53, while miR-6775-3p abrogated the inhibition of p53 transcription activity through targeting MAGE-A family.

### miR-6775-3p and its host gene SLC7A5 are directly regulated by p53

**Fig. 6:**
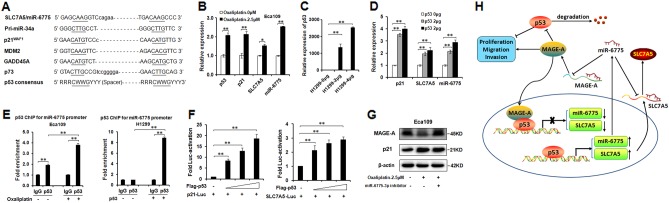
**miR-6775 and its host gene SLC7A5 are directly regulated by p53.** **a** Alignment of the putative SLC7A5/miR-6775 p53 responsive element with known p53 responsive elements. **b** Expression of p53, p21, SLC7A5, miR-6775-3p in Eca109 cells after treatment with 2.5 μM of Oxaliplatin, detected by qRT-PCR. **c** Transfection efficiency of p53 expression plasmid in H1299 cells detected with qRT-PCR. **d** Expression of p21, SLC7A5, miR-6775-3p in Eca109 cells after transfected with p53 expression plasmid, detected by qRT-PCR. **e** ChIP results showed the binding of p53 on the promoter of SLC7A5/miR-6775 in Eca109 cells treated with 2.5 μM of Oxaliplatin and in H1299 cells transfected with p53 expression plasmid. **P < 0.01. **f** Luciferase reporter assays for luciferase activity of luc-p21^WAF1^ and SLC7A5/miR-6775 promoter in H1299 cells co-transfected with combination of different plasmids. **P < 0.01. **g** Expression of MAGE-A, p21 in Eca109 cells after treatment with 2.5 μM of Oxaliplatin and miR-6775-3p inhibitor, detected by western blot. **h** Schematic model of the feedback loop between miR-6775-3p and p53 via MAGE-A proteins

Some studies reported that intronic miRNAs had the common regulatory mechanisms and expression patterns with their host gene^14,15^. miR-6775-3p was processed from the intron of its host gene SLC7A5. To determine how miR-6775-3p is regulated in cancer progression, we analyzed the promoter of SLC7A5, and found that there exists one p53 binding site on the −1362 bp upstream of SLC7A5 from the transcription start site (Fig 6a). To verify if SLC7A5 and miR-6775-3p is regulated by p53, p53 wild-type Eca109 cells were treated with 2.5 μM of Oxaliplatin, and the expression of p53, p21, SCL7A5 and miR-6775-3p were detected. qRT-PCR results showed that p53 is up-regulated upon Oxaliplatin treatment, and p21, SLC7A5 and miR-6775-3p were also increased after Oxaliplatin treatment in both Eca109 cells (Fig. 6b). We then transfected Flag-p53 expression plasmid into p53-deficient H1299 cells, and qRT-PCR results showed the up-regulation of p21, SLC7A5 and miR-6775 in p53 over-expressed cells (Fig. 6c, d). In order to determine whether SLC7A5 and miR-6775-3p are direct transcriptional targets of p53, we performed p53 ChIP for SLC7A5 promoter in Eca109 cells treated with or without 2.5 μM of Oxaliplatin. ChIP results showed that the specific binding of p53 to SLC7A5 promoter was increased as compared with IgG control in Eca109 cells. When cells treated with Oxaliplatin, the specific binding of p53 to SLC7A5 promoter was markedly increased as compared with the untreated cells (Fig. 6e). In p53-deficient H1299 cells, enforced expression of p53 enhanced the specific binding of p53 to SLC7A5 promoter as compared with IgG control (Fig. 6e). In addition, the luciferase reporter assay results demonstrated that p53 increased the luciferase reporter activity of p21^WAF1^ and SLC7A5/miR-6775 promoters in a dose dependent manner (Fig 6f). In Eca109 cells, we found that the activation of p53 after treatment with 2.5 μM of Oxaliplatin led to MAGE-A protein downregulation, but no significant change when added miR-6775-3p inhibitor at the same time (Fig 6g). Taken together, our results suggested that p53 directly regulates miR-6775-3p and its host gene SLC7A5.

### miR-6775-3p directly targets its host gene SLC7A5

Interestingly, we found that there exists three miR-6775-3p binding site on the 3′UTR region of SLC7A5 (Supplementary Figure S3A). For evaluate the effect of miR-6775-3p on the expression of SLC7A5, we performed qRT-PCR and western blot analysis. Our results showed that SLC7A5 expression was repressed in miR-6775-3p-overexpressed TE1 cells, and increased in miR-6775-3p-suppressed Ec9706 cells (Supplementary Figure S3B, S3C). We also found the decreased SLC7A5 expression in miR-6775-3p agomir-transfected TE1 mice xenograft tissues (Supplementary Figure S3D). A negative correlation between miR-6775-3p and SLC7A5 expression was found in human ESCC tissues (Supplementary Figure S3E). These results suggested that miR-6775-3p directly targets its host gene SLC7A5.

## Discussion

Accumulating evidence demonstrated that miRNAs play critical roles in human cancer progression^23–26^. In the present study, we firstly found that miR-6775-3p plays tumor suppressive role in ESCC progression based on the following observations: (1) ESCC patients with higher expression of miR-6775-3p had a better clinical prognosis; (2) Enforced expression of miR-6775-3p inhibited tumor growth and liver metastasis of ESCC xenograft; (3) Over-expression of miR-6775-3p inhibited cell proliferation, migration, and invasion of ESCC cells, while downregulation of miR-6775-3p increased proliferation, migration and invasion of ESCC cells; (4) miR-6775-3p directly targeted tumor antigens MAGE-A family which plays oncogenic roles in cancer progression; (5) miR-6775-3p attenuated MAGE-A-inhibited transcriptional activity of p53; (6) miR-6775-3p directly targeted its host gene SLC7A5 which has been reported to play oncogenic roles in cancer progression^27–29^. In addition, KEGG pathway analysis revealed that miR-6775-3p was associated with the “pathway in cancer”. Most of the target genes of miR-6775-3p are shown to play oncogenic roles in cancer progression. Our data suggested that miR-6775-3p might be a potential tumor repressive miRNA.

The regulation mechanism of miRNAs is still not fully elucidated. Transcription of miRNAs are likely to be regulated by the same mechanisms that govern mRNA expression. As a transcription factor, tumor suppressor p53 drives the expression of a large number of genes in response to multiple stimuli. Over the past decade, a number of miRNAs have been identified as new p53 targets^30–35^. Interestingly, a feedback regulation between p53 and its target miRNAs was also found, positively or negatively. For instance, miR-605 and miR-145 were found to be transcriptionally induced by p53 to suppress MDM2 through binding its 3′UTR, thus protecting p53 from MDM2-mediated degradation^36–38^. These two miRNAs form a positive feedback regulation with p53 via MDM2. In addition, miR-29 was also reported to be transcriptionally induced by p53 in response to DNA damage, and positively influence p53 level by repressing the expression of PPM1D phosphatase that dephosphorylates p53 and destabilizes p53, therefore forming a positive feedback loop with p53 in response to DNA damage^39,40^.These studies revealed that some p53-targeted miRNAs modulate p53 expression and activity through forming a feedback regulation pattern.

In our present study, we found that p53 directly transcriptionally induce the expression of miR-6775-3p and its host gene SLC7A5. miR-6775-3p inhibited the expression of tumor antigens MAGE-A family by directly binding the 3′UTR region of MAGE-A mRNAs. MAGE-A family proteins have been demonstrated to play functions in cells through two aspects including as regulator of E3 RING ubiquitin ligases and as transcription regulators, through which MAGE-A family inhibit p53 transcription activity and increase p53 degradation^19–21,41–45^. In our study, miR-6775-3p attenuated MAGE-A-inhibited transcriptional activity of p53. Thus, miR-6775-3p modulates p53 activity through forming a positive feedback regulation via MAGE-A family. Interestingly, miR-6775-3p also inhibited its host gene SLC7A5 through direct binding the 3′UTR region of SLC7A5 mRNA. SLC7A5 has been demonstrated to play an oncogenic role in cancer progression. Therefore, miR-6775-3p plays its tumor suppressive function at least in part through inhibiting its host gene SLC7A5 after transcribed by p53.

In conclusion, our founding suggested a novel positive feedback regulation between miR-6775-3p and p53 via MAGE-A family proteins, which plays critical role in ESCC progression. In addition, miR-6775-3p functions as a tumor suppressive miRNA through in part through inhibiting its host gene SLC7A5. Our findings provide a new therapeutic target for ESCC treatment.

## Novelty and impact

For the first time, we demonstrated miR-6775-3p plays a tumor suppressive role in ESCC through targeting tumor antigens MAGE-A family. miR-6775-3p inhibited MAGE-A family expression through binding their 3′UTR regions, and attenuated MAGE-A-inhibited transcriptional activity of p53. miR-6775-3p also directly inhibits its host gene SLC7A5. Interestingly, miR-6775-3p and SLC7A5 were directly transcriptionally induced by p53. Thus, we proposed a novel positive feedback regulation between miR-6775-3p and p53 via MAGE-As family in ESCC.

## Electronic supplementary material

Supplementary Tables

miR-6775-3p expression in ESCC cell lines

MAGE-A9 rescue miR-6775-3p

miR-6775-3p target SLC7A5

Supplementary Figure Legends
