# Supplementary material for: Tumor suppressive miR-6775-3p inhibits ESCC progression through forming a positive feedback loop with p53 via MAGE-A family proteins
Source: Cell Death Dis. 2018 Oct 17;9(11):1057. doi: 10.1038/s41419-018-1119-3 (PMC6193014; doi:10.1038/s41419-018-1119-3)
Supplement: Supplementary file 1 — Supplementary Tables [file 41419_2018_1119_MOESM1_ESM.docx]

**Supplementary Table S1 The primer and miR-6775-3p sequences**

| **Genes** | **Primers** |
| --- | --- |
| ***MAGE-A*** | forward, 5’- CCTGACCTGATAGACCCTG -3’  reverse, 5’-AGCATTTCTGCCTTTGTG-3’ |
| ***P53*** | forward, 5’-GCTCAAGACTGGCGCTAAAA-3’  reverse, 5’-GTGTCACCGTCGTGGAAAG-3’ |
| ***P21*** | forward, 5’-CAACATTTTCGGCAGCTAAAC-3’  reverse, 5’-CAGCCAGCTTGAAGGAACC-3’ |
| ***SLC7A5*** | forward, 5’-CTGCTCAAGCCGCTCTTC-3’  reverse, 5’-CACGCTGTAGCAGTTCACG-3’ |
| ***GAPDH*** | forward, 5’-AGCCACATCGCTCAGACAC-3’  reverse, 5’-GCCCAATACGACCAAATCC-3’ |
| **Has-miR-6775-3p agomir** | Sense, 5’ -AGGCCCUGUCCUCUGCCCCAG- 3’ chol  Antisense,5’-GGGGCAGAGGACAGGGCCUUU-3’ chol |
| **Has-miR-6775-3p mimics** | Sense, 5’ -AGGCCCUGUCCUCUGCCCCAG- 3’  Antisense,5’ -GGGGCAGAGGACAGGGCCUUU-3’ |
| **Has-miR-6775-3p inhibitor** | 5’-CUGGGGCAGAGGACAGGGCCU-3’ |

**Supplementary Table S2 Correlation between miR-6775-3p expression and the clinicopathological parameters of ESCC patients**

| **Variables** | **Cases** | **miR-6775-3p** | | |
| --- | --- | --- | --- | --- |
|  |  | **Low** | **High** | ***P*** |
| **Age(y/s)** |  |  |  | 0.878 |
| **≤55** | 62 | 31 | 31 |  |
| **>55** | 76 | 39 | 37 |  |
| **Gender** |  |  |  | 0.305 |
| **Male** | 93 | 50 | 43 |  |
| **Female** | 45 | 20 | 25 |  |
| **Histological grade** |  |  |  | 0.044 |
| **G1** | 12 | 8 | 4 |  |
| **G2** | 85 | 36 | 49 |  |
| **G3-4** | 41 | 26 | 15 |  |
| **Tumor infiltration** |  |  |  | 0.031 |
| **T1-2** | 87 | 38 | 49 |  |
| **T3-4** | 51 | 32 | 19 |  |
| **Lymph node metastasis** |  |  |  | 0.005 |
| **N0** | 19 | 4 | 15 |  |
| **N1** | 118 | 66 | 53 |  |
| **Distant metastasis** |  |  |  | 0.004 |
| **M0** | 98 | 42 | 56 |  |
| **M1-lym*** | 40 | 28 | 12 |  |

* Distant lymph node or distant organ metastasis

**Supplementary Table S3 Significantly enriched annotations regulated by miR-6775-3p in TargetScan database**

| No. Genes | P value | Annotations |
| --- | --- | --- |
| 63 | 0.001156 | Pathways in cancer |
| 51 | 0.004622 | MAPK signaling pathway |
| 41 | 0.008642 | Regulation of actin cytoskeleton |
| 29 | 0.002163 | Neurotrophin signaling pathway |
| 22 | 0.002953 | Dilated cardiomyopathy |
| 22 | 0.000957 | Hypertrophic cardiomyopathy (HCM) |
| 20 | 0.009228 | ErbB signaling pathway |
| 19 | 0.003087 | Arrhythmogenic right ventricular cardiomyopathy (ARVC) |
| 17 | 0.000857 | Acute myeloid leukemia |
| 17 | 0.000427 | Non-small cell lung cancer |
| 17 | 0.004075 | Glioma |
| 17 | 0.009037 | Renal cell carcinoma |
| 14 | 0.006671 | mTOR signaling pathway |
| 9 | 0.001252 | Glycosaminoglycan biosynthesis-chondroitin sulfate/dermatan sulfate |
