# Supplementary figures and images for: Tumor suppressive miR-6775-3p inhibits ESCC progression through forming a positive feedback loop with p53 via MAGE-A family proteins
Source: Cell Death Dis. 2018 Oct 17;9(11):1057. doi: 10.1038/s41419-018-1119-3 (PMC6193014; doi:10.1038/s41419-018-1119-3)

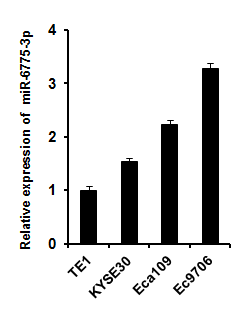

Supplement: Supplementary file 2 — miR-6775-3p expression in ESCC cell lines [file 41419_2018_1119_MOESM2_ESM.tif]

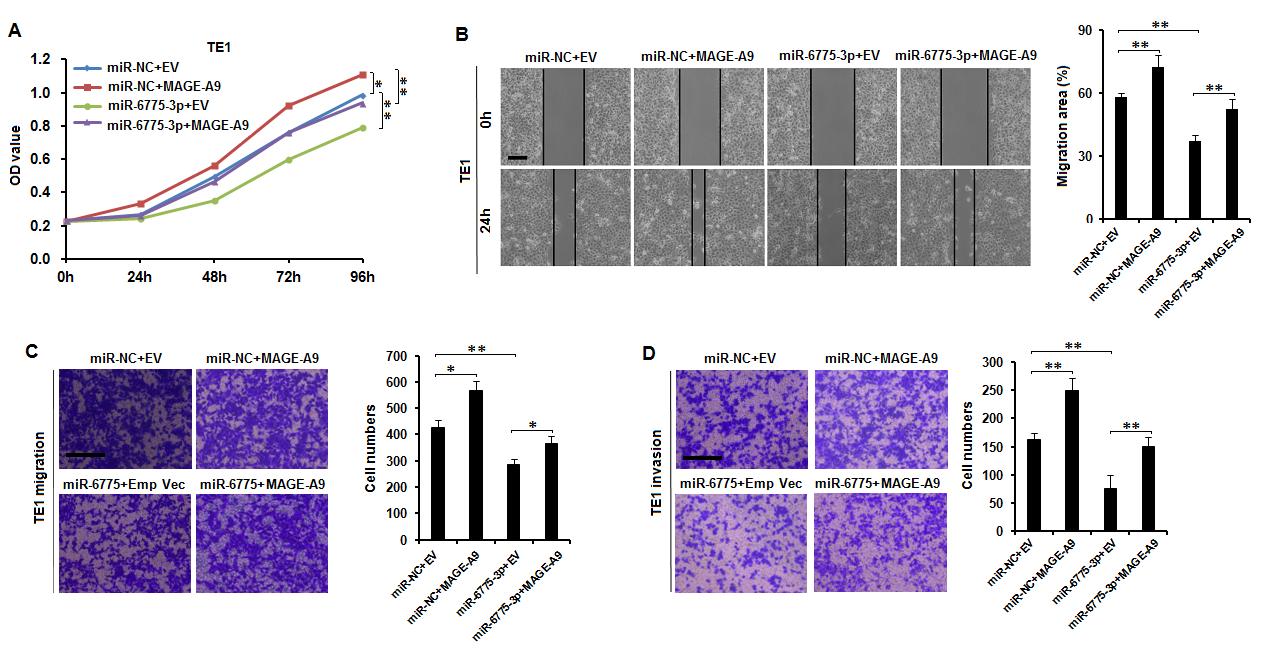

Supplement: Supplementary file 3 — MAGE-A9 rescue miR-6775-3p [file 41419_2018_1119_MOESM3_ESM.tif]

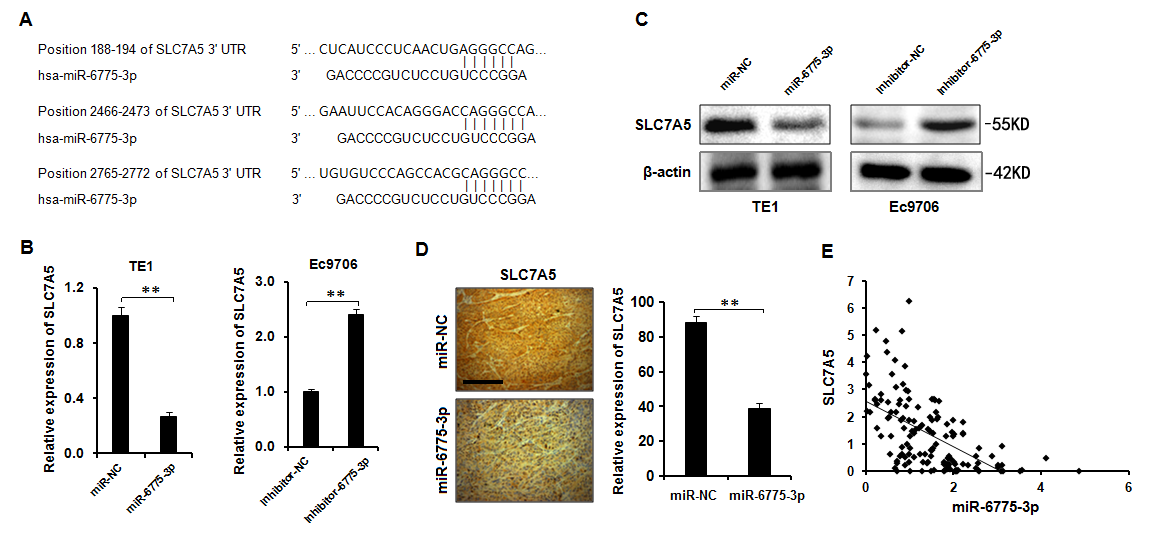

Supplement: Supplementary file 4 — miR-6775-3p target SLC7A5 [file 41419_2018_1119_MOESM4_ESM.tif]
