# Supplementary material for: Tumor suppressive miR-6775-3p inhibits ESCC progression through forming a positive feedback loop with p53 via MAGE-A family proteins
Source: Cell Death Dis. 2018 Oct 17;9(11):1057. doi: 10.1038/s41419-018-1119-3 (PMC6193014; doi:10.1038/s41419-018-1119-3)
Supplement: Supplementary file 5 — Supplementary Figure Legends [file 41419_2018_1119_MOESM5_ESM.doc]

**Supplementary Figure Legends**

**Supplementary Figure S1.** The expression of miR-6775-3p in four ESCC cell lines.

**Supplementary Figure S2.** miR-6775-3p inhibits the proliferation, migration and invasion of ESCC cells by direct targeting MAGE-As. A, MTT assay showed that miR-6775-3p suppressed cell proliferation of TE1 cells. After co-transfeced with miR-6775-3p mimics and MAGE-A9, the suppressive effect of miR-6775-3p was reversed. **P*<0.05, ***P*<0.01. B, Wound healing experiment showed that miR-6775-3p suppressed the migration of TE1 cells. After co-transfeced with miR-6775-3p mimics and MAGE-A9, the suppressive effect of miR-6775-3p was reversed. **P*<0.05. Bars: 50μm. C and D, Transwell migration and matrigel invasion assay showed that miR-6775-3p suppressed cell migration and invasion abilities of TE1 cells. After co-transfeced with miR-6775-3p mimics and MAGE-A9, the suppressive effect of miR-6775-3p was reversed. Bars: 100 μm. **P*<0.05, ***P*<0.01.

**Supplementary Figure S3.** miR-6775-3p directly targets its host gene SLC7A5. A, The binding sites of miR-6775-3p with the 3’UTR of SLC7A5. B, Expression of SLC7A5 in TE1 cells transfected with miR-6775-3p mimics or miR-NC, and in Ec9706 cells transfected with miR-6775-3p inhibitor or inhibitor-NC, detected by qRT-PCR. ***P*<0.01. C, Expression of SLC7A5 in TE1 cells transfected with miR-6775-3p mimics or miR-NC and Ec9706 cells transfected with miR-6775-3p inhibitor or inhibitor-NC, detected by western blot. D, The expression of SLC7A5 in mice tumor tissues was examined by IHC. Bars: 50 μm. ***P*<0.01. G, Expression correlation between miR-6775-3p and SLC7A5 detected by qRT-PCR.
